# Varying optimal power for height-standardisation of childhood weight, fat mass and fat-free mass across the obesity epidemic

**DOI:** 10.1038/s41366-024-01619-y

**Published:** 2024-09-03

**Authors:** Mohammed T. Hudda, Julie Aarestrup, Christopher G. Owen, Jennifer L. Baker, Peter H. Whincup

**Affiliations:** 1 Department of Population Health, Dasman Diabetes Institute, Kuwait City, Kuwait; 2 Center for Clinical Research and Prevention, Copenhagen University Hospital—Bispebjerg and Frederiksberg, Copenhagen, Denmark; 3 Population Health Research Institute, City St George’s, University of London, London, UK

**Keywords:** Epidemiology, Risk factors

## Abstract

**Introduction:**

Childhood adiposity markers can be standardised for height in the form of indices (marker/height^p^) to make meaningful comparisons of adiposity patterns within and between individuals of differing heights. The optimal value of p has been shown to differ by birth year, sex, age, and ethnicity. We investigated whether height powers for childhood weight and fat mass (FM) differed by birth year, sex, or age over the period before and during the child obesity epidemic in Copenhagen.

**Setting/methods:**

Population-based cross-sectional study of 391,801 schoolchildren aged 7 years, 10 years and 13 years, born between 1930 and 1996, from the Copenhagen School Health Records Register. Sex- and age-specific estimates of the height powers for weight and FM were obtained using log–log regression, stratified by a decade of birth.

**Results:**

For weight, amongst children born 1930–39, optimal height powers at 7 years were 2.20 (95% CI: 2.19–2.22) for boys and 2.28 (95% CI: 2.26–2.30) for girls. These increased with birth year to 2.82 (95% CI: 2.76–2.87) and 2.92 (95% CI: 2.87–2.97) for boys and girls born in 1990–96, respectively. For FM, amongst those born 1930–39, powers at 7 years were 2.46 (95% CI: 2.42–2.51) and 2.58 (95% CI: 2.53–2.63) for boys and girls, respectively, and increased with birth year reaching 3.89 (95% CI: 3.75–4.02) and 3.93 (95% CI: 3.80–4.06) for boys and girls born 1990–96, respectively. Powers within birth cohort groups for weight and FM were higher at 10 years than at 7 years, though similar increases across groups were observed at both ages. At 13 years, height powers for weight and FM initially increased with the birth year before declining from the 1970s/80s.

**Conclusion:**

Due to increases in the standard deviation of weight and FM during the obesity epidemic, optimal height powers needed to standardise childhood weight and FM varied by birth year, sex, and age. Adiposity indices using a uniform height power mean different things for different birth cohort groups, sexes, and ages thus should be interpreted with caution. Alternative methods to account for height in epidemiological analyses are needed.

## Background

Childhood overweight and obesity pose a major global public health challenge with ~18% of all children and adolescents aged 5–19 years worldwide (i.e. over 340 million individuals) estimated as living with overweight or obesity [1]. The World Health Organisation defines obesity in terms of the health risks associated with “excessive fat accumulation” [2]. However, in the absence of direct techniques to quantify body fat historically, childhood adiposity assessments (and classifications of overweight and obesity) have been widely based upon body mass index (BMI; weight/height^2^), a surrogate marker of adiposity based on total body weight after standardisation for height. However, it has been demonstrated that while adopting a height power of two used in the derivation of BMI may be practical due to the ease of calculation, the power term is too low in childhood, thus providing poor height standardisation [3–6]. An alternative approach to adopting the power term two is to develop a weight-for-height index of the form, weight/height^*p*^, where p is the optimal height power needed to remove the dependence of weight on height [7, 8] and is obtained from a general linear regression of log-transformed weight (kg) on log-transformed height (m). Obtaining a consistent optimal value of p to standardise childhood weight is complex and the optimal value of p has been shown to differ by sex, age and ethnicity [3–6, 9]. This is unsurprising given *p*, is equal to

$$p=r\frac{{SD\; of\; log}({weight})}{{SD\; of\; log}({height})}$$

where $r$ is the correlation coefficient between log-transformed weight and log-transformed height [6, 10]. Therefore, variation in weight and height and the correlation between them will influence the optimal value of *p*. A previous study conducted in four British birth cohorts from 1946, 1958, 1970, and 2001 found that while the optimal height power decreased with age across the life course, the height power needed to standardise childhood weight for 11-year-old was higher in the more recent birth cohorts compared to within earlier cohorts [6]. It would therefore be of value to further investigate changes in the height powers over the period before and during the child obesity epidemic for a range of childhood ages in other childhood populations.

Additionally, given the availability of several techniques to assess childhood fat mass (FM) and fat-free mass (FFM) more directly, there is an argument to shift the focus away from weight-based proxy markers, and towards FM assessment. This is particularly important given the results of our earlier study which demonstrated that childhood FM was more strongly associated with adult type 2 diabetes risk than weight-based markers, after adjustment for height [11]. While the use of FM and FFM in research studies has become increasingly popular, several studies have simply adopted a height power of two to standardise FM and FFM for height [12–14], as this is the most common power used in the standardisation of childhood weight to form BMI and thus retaining the same scale (kg/m^2^) of the markers. This raises the question of whether optimal height powers to standardise FM and FFM in childhood exist, or whether these power terms differ by birth year, sex, age or ethnicity.

We therefore aimed to investigate whether optimal height powers for standardising weight, FM and FFM amongst 7-, 10- and 13-year-old schoolchildren from Copenhagen, Denmark were consistent over the birth year period from 1930 to 1996, and by sex and age.

## Methods

### Study population

This study was based on The Copenhagen School Health Records Register (CSHRR), a database containing information on almost every schoolchild in Copenhagen born between 1930 and 1996 [15]. The cohort members had regular health examinations including height and weight measurements, which were recorded by school-based doctors or nurses [15]. Ethnicity information was consistently recorded in the CSHRR database for children born from 1990 onwards. This study included all individuals with complete information on height, weight, age, and sex for at least one childhood measurement at 7 years, 10 years, or 13 years of age.

### Statistical analysis

#### Prediction of childhood FM and FFM

STATA version 17 was used for all analyses. Predictions of childhood FFM (and FM by subtraction from measurements of weight) within the CSHRR were made using an open-access and extensively validated prediction equation which is based on information on height, weight, age, sex, and ethnicity [16–18]. Extensive external validations of this equation both in the UK [16, 18] and in 19 childhood settings globally [17] demonstrated its high accuracy for estimating childhood FM and FFM with a pooled *R*^2^, calibration slope, and calibration-in-the-large values (95% confidence intervals) across all settings of 88.7% (85.9% to 91.4%), 0.98 (0.97 to 1.00), and 0.01 (−0.02 to 0.04), respectively, and with a root mean square error value of <4 kg in 17 of the 19 countries [17]. Additionally, the prediction equation has been shown to produce estimates of body composition more accurately than those provided by bioelectrical impedance and DXA [18], which are widely used in clinical and research settings to estimate body composition in childhood. Where consistent information on the ethnic origins of the children in the CHSRR was not available (i.e. in the earlier years of the cohort), these children were assumed to be of White European origins for the purpose of estimating FM as the proportion of children from a non-White (European) ethnic background was low owing to migration patterns to Denmark at this time [15].

Key variables of weight, FM, FFM, and height were summarised in terms of their median and inter-quartile range (IQR) by sex and age, within each birth cohort group (1930–39, 1940–49, 1950–59, 1960–69, 1970–79, 1980–89, and 1990–96). In addition, given the formula used to calculate the optimal power is based on the correlations between the log-transformed body size markers, log-transformed height and their respective standard deviations (SDs), we also estimated these sex- and age-specific parameters within each birth cohort group.

#### Estimation of the optimal height powers for weight, FM and FFM

The optimal power was estimated for weight, FM, and FFM by regressing each log-transformed marker on log-transformed height, adjusting for exact age in months. Sex- and age-stratified models were first fitted within the birth cohort group to obtain group-specific cross-sectional estimates of the power, and then re-fitted across all birth cohort groups, adjusting for birth cohort, to obtain an overall average cross-sectional estimate across all groups. Sex- and age-specific correlation coefficients were quantified across birth cohort groups and overall, between height and weight, FM, and FFM indices (each derived using a range of height powers).

Finally, in the most recent birth cohort group (born 1990–96) where information on the ethnic origins was consistently available, we explored ethnic patterns in the key variables, estimated the sex- and age-specific correlation coefficients between log-transformed body size markers and log(height), and estimated sex- and age-specific height powers for three ethnic groups, White, Black, and South Asians.

Supplementary analyses were conducted to formally test for differences in the cross-sectional estimates of the optimal height powers for weight, FM and FFM by birth cohort, age, and sex. A multi-level regression model was fitted, combining all birth cohort groups, ages and both sex groups, with the log-transformed marker as the outcome of the model and including fixed effects terms of log-transformed height, sex, age (in months), and birth cohort group and with a random effects term for individual ID. We then included and tested the statistical significance (using a 1% significance level to account for multiple testing) of interaction terms between: (i) log-transformed height and age, (ii) log-transformed height and sex, (iii) log-transformed height and birth cohort group, and (iv) log-transformed height, age, sex, and birth cohort group.

## Results

Childhood measurements of weight, height, and predicted FM and FFM were available across the seven birth-cohort groups in 355,310 children aged 7 years (181,382 boys and 173,928 girls), 323,758 children aged 10 years (162,244 boys and 161,514 girls), and 310,888 children aged 13 years (154,269 boys and 156,619 girls). Median levels and IQRs for each body size variable at 7 years, 10 years, and 13 years of age in boys and girls, by birth cohort group and overall, are presented in Tables 1 and 2. Median and IQRs of FM, FFM, weight and height generally increased with birth cohorts for all three childhood ages and among both sexes (Tables 1 and 2). Average levels of FM and weight were higher for boys and girls born in the 1990s compared with those born in the 1930s. For example, amongst boys, average FM and weight were higher by 26% and 13%, respectively for 7-year-olds, by 32% and 15%, respectively for 10-year-olds, and by 35% and 25%, respectively for 13-year-olds (Table 1). Median height amongst 7-year-old boys born in the 1990s was 7% higher compared with those born in the 1930s (Table 1). Similar percentage increases in average levels of FM, weight and height were observed amongst girls with the exception of 10-year-olds, where increases in average height across the birth cohort groups were higher than those observed for boys, and at 13-year-olds where percentage increases in all three markers were less than those observed for boys (Table 2).

**Table 1:** Median (lower quartile–upper quartile) of body size variables amongst boys aged 7 years, 10 years, and 13 years, by birth cohort group.

		Birth cohort group						
Age								
	Body size marker	1930–39	1940–49	1950–59	1960–69	1970–79	1980–89	1990–96
7 Years		*N* = 38,410	*N* = 52,500	*N* *=* 31,736	*N* = 20,890	*N* = 14,437	*N* = 12,489	*N* = 10,920
	Fat mass (kg)	4.50 (3.88–5.30)	4.88 (4.15–5.77)	4.85 (4.11–5.77)	4.97 (4.21–5.94)	5.26 (4.44–6.32)	5.43 (4.51–6.68)	5.67 (4.67–7.05)
	Fat-free mass (kg)	18.39 (17.17–19.70)	19.11 (17.84–20.46)	19.21 (17.94–20.64)	19.56 (18.24–21.00)	19.86 (18.51–21.33)	20.12 (18.67–21.71)	20.22 (18.72–21.86)
	Weight (kg)	23.0 (21.2–25.0)	24.0 (22.1–26.1)	24.0 (22.1–26.3)	24.5 (22.5–26.9)	25.0 (23.0–27.5)	25.5 (23.2–28.3)	26.0 (23.5–28.9)
	Height (m)	1.23 (1.19–1.26)	1.25 (1.21–1.28)	1.25 (1.22–1.29)	1.26 (1.22–1.30)	1.26 (1.23–1.30)	1.27 (1.23–1.31)	1.27 (1.24–1.31)
10 Years		*N* = 38,838	*N* = 53,918	*N* = 31,478	*N* = 21,157	*N* = 7896	*N* = 5366	*N* = 3591
	Fat mass (kg)	6.51 (5.47–7.78)	6.98 (5.79–8.59)	6.85 (5.63–8.53)	6.98 (5.74–8.67)	7.41 (5.94–9.47)	8.14 (6.27–11.37)	8.60 (6.61–12.15)
	Fat-free mass (kg)	24.63 (22.94–26.53)	25.67 (23.83–27.73)	25.81 (23.94–28.00)	26.29 (24.36–28.50)	26.70 (24.59–29.10)	27.39 (24.99–30.24)	27.50 (24.99–30.46)
	Weight (kg)	31.2 (28.6–34.1)	32.7 (30.0–36.1)	32.7 (29.9–36.4)	33.3 (30.3–37.0)	34.0 (30.9–38.5)	35.5 (31.5–41.5)	36.0 (32.0–42.2)
	Height (m)	1.38 (1.34–1.42)	1.40 (1.36–1.44)	1.41 (1.37–1.45)	1.42 (1.38–1.46)	1.42 (1.38–1.47)	1.43 (1.39–1.48)	1.43 (1.39–1.48)
13 Years		*N* = 35,292	*N* = 52,288	*N* = 32,068	*N* = 19,892	*N* = 4566	*N* = 5110	*N* = 5053
	Fat mass (kg)	8.01 (6.35–10.08)	8.62 (6.71–11.01)	8.17 (6.28–10.72)	8.49 (6.55–10.99)	9.10 (6.79–12.71)	10.21 (7.57–14.24)	10.79 (7.82–15.02)
	Fat-free mass (kg)	33.92 (30.89–37.56)	35.79 (32.37–39.85)	36.06 (32.50–40.38)	37.12 (33.34–41.68)	38.08 (33.77–43.47)	40.35 (36.01–45.31)	41.19 (36.50–46.11)
	Weight (kg)	42.0 (37.7–47.5)	44.6 (39.7–50.7)	44.5 (39.3–51.0)	46.0 (40.5–52.5)	47.7 (41.1–56.0)	51.1 (44.5–59.0)	52.6 (45.5–60.9)
	Height (m)	1.53 (1.48–1.59)	1.56 (1.51–1.62)	1.57 (1.51–1.63)	1.59 (1.53–1.65)	1.60 (1.53–1.66)	1.62 (1.56–1.69)	1.63 (1.57–1.70)

**Table 2:** Median (lower quartile–upper quartile) of body size variables amongst girls aged 7 years, 10 years, and 13 years, by birth cohort group.

		Birth cohort group						
Age								
	Body size marker	1930–39	1940–49	1950–59	1960–69	1970–79	1980–89	1990–96
7 Years		*N* = 34,385	*N* = 51,234	*N* = 31,162	*N* = 20,928	*N* = 14,070	*N* = 11,728	*N* = 10,421
	Fat mass (kg)	5.24 (4.51–6.16)	5.55 (4.73–6.58)	5.54 (4.73–6.63)	5.63 (4.78–6.79)	5.98 (5.03–7.21)	6.20 (5.14–7.74)	6.45 (5.29–8.21)
	Fat-free mass (kg)	17.30 (16.09–18.60)	17.85 (16.64–19.22)	17.99 (16.74–19.39)	18.31 (17.00–19.70)	18.61 (17.31–20.09)	18.89 (17.50–20.52)	18.95 (17.45–20.61)
	Weight (kg)	22.5 (20.7–24.7)	23.4 (21.5–25.7)	23.5 (21.5–26.0)	24.0 (22.0–26.5)	24.5 (22.5–27.2)	25.0 (22.7–28.0)	25.5 (23.0–28.7)
	Height (m)	1.22 (1.18–1.26)	1.24 (1.20–1.27)	1.24 (1.21–1.28)	1.25 (1.22–1.29)	1.26 (1.22–1.29)	1.26 (1.22–1.30)	1.26 (1.23–1.30)
10 Years		*N* = 37,937	*N* = 54,080	*N* = 31,304	*N* = 21,512	*N* = 7936	*N* = 5240	*N* = 3505
	Fat mass (kg)	7.62 (6.40–9.23)	8.10 (6.65–10.12)	8.03 (6.55–10.13)	8.13 (6.59–10.27)	8.52 (6.89–11.11)	9.47 (7.19–13.08)	10.00 (7.53–14.06)
	Fat-free mass (kg)	23.28 (21.57–25.20)	24.23 (22.36–26.36)	24.47 (22.56–26.70)	24.95 (22.93–27.24)	25.45 (23.25–28.00)	26.21 (23.61–29.38)	26.43 (23.58–29.52)
	Weight (kg)	31.0 (28.2–34.3)	32.4 (29.2–36.4)	32.5 (29.4–36.7)	33.0 (29.8–37.5)	34.0 (30.4–39.0)	35.8 (31.0–42.3)	36.5 (31.5–43.7)
	Height (m)	1.37 (1.33–1.42)	1.39 (1.35–1.44)	1.40 (1.36–1.45)	1.42 (1.37–1.46)	1.43 (1.38–1.47)	1.43 (1.38–1.48)	1.44 (1.39–1.49)
13 Years		*N* = 36,734	*N* = 52,418	*N* = 32,417	*N* = 20,115	*N* = 4529	*N* = 5237	*N* = 5169
	Fat mass (kg)	10.93 (8.70–13.71)	11.24 (8.87–14.09)	11.01 (8.57–13.98)	11.33 (8.82–14.37)	12.02 (9.06–15.86)	13.16 (9.82–17.18)	13.06 (9.93–17.36)
	Fat-free mass (kg)	33.96 (31.01–36.93)	35.30 (32.34–38.28)	35.72 (32.68–38.77)	36.49 (33.41–39.69)	37.09 (33.62–40.56)	37.84 (34.63–41.36)	37.97 (34.76–41.53)
	Weight (kg)	45.0 (40.2–50.2)	46.7 (41.8–52.0)	47.0 (41.9–52.4)	48.0 (42.8–53.8)	49.3 (43.5–56.0)	51.0 (45.2–58.0)	51.2 (45.5–58.1)
	Height (m)	1.55 (1.50–1.60)	1.57 (1.53–1.62)	1.58 (1.54–1.63)	1.60 (1.55–1.64)	1.60 (1.55–1.65)	1.61 (1.56–1.65)	1.61 (1.57–1.66)

Histograms of weight, FM, FFM and height at each of the three childhood ages, stacked by birth cohort group, demonstrate the shift in the distribution of these variables over time, and show the increasing positive skewed distributions for both weight and FM for boys and girls (Supplementary Figs. 1 and 2). Sex-specific SDs of weight, FM, FFM, and height at each of the three ages within each birth cohort group are presented in Supplementary Tables 1 and 2. While the SDs of height were fairly constant across the birth cohort groups for both boys and girls, the SDs of weight, FM and FFM increased with birth year, with the SDs of FM showing the greatest increases across the birth cohort group (Supplementary Tables 1 and 2). Sex- and age-specific correlation coefficients between log(weight), log(FM), log(FFM) and log(height), by birth cohort group, are presented in Supplementary Tables 3 and 4. Correlations between log(FM) and log(height) at 7- and 10-years, amongst both boys and girls, were generally stable across the birth cohort groups but amongst 13-year-old boys and girls, correlations were weaker in later birth cohort groups compared to earlier cohorts. The correlations between log(height) and both log(FFM) and log(weight) for boys and girls at each age were weaker in later birth cohort groups compared to earlier cohort groups (Supplementary Tables 3 and 4).

### Estimation of the optimal height powers for weight, FM, and FFM

**Fig. 1:**
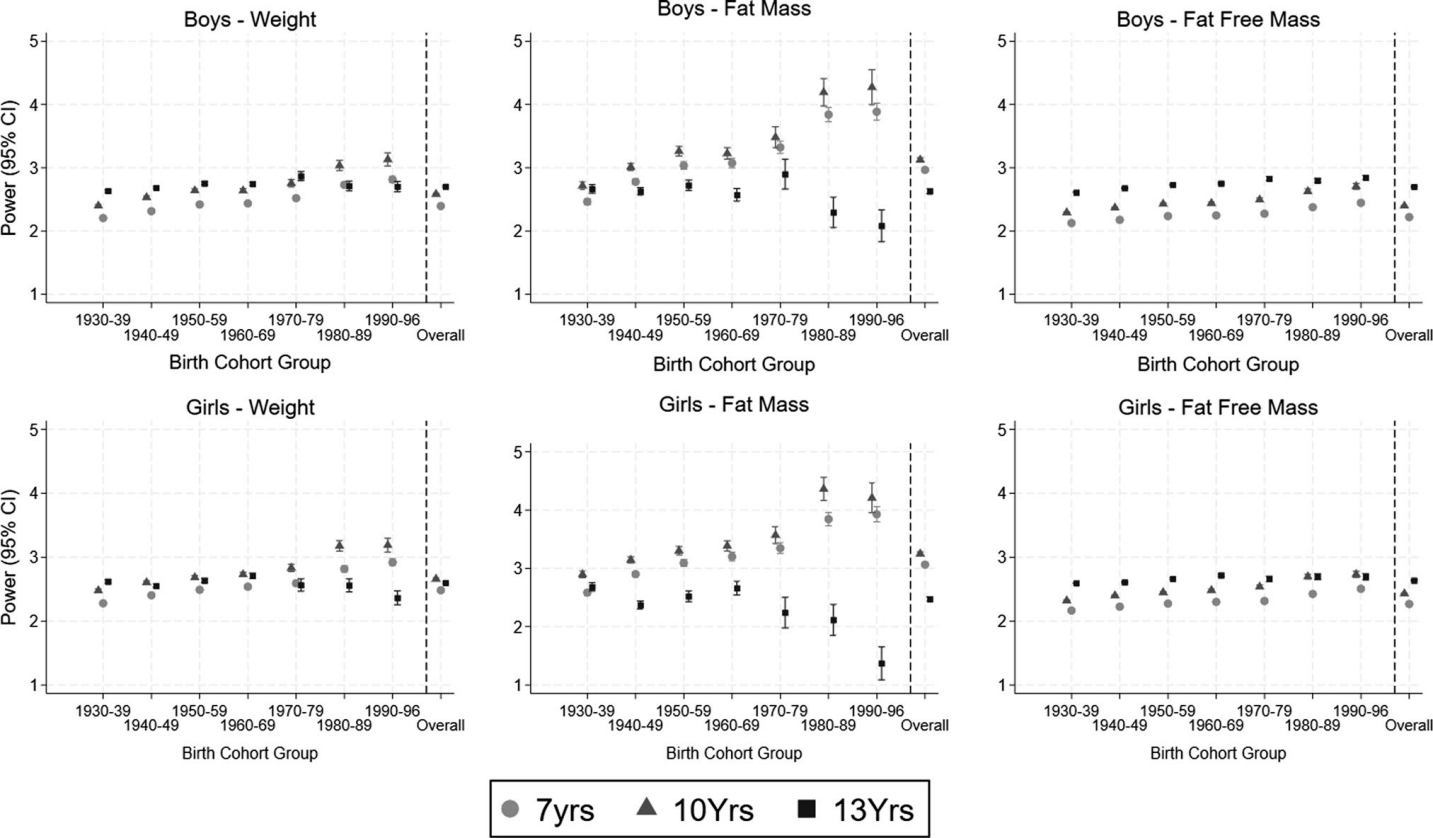
Sex- and age-specific height powers to standardise body size variables for height, by birth cohort and overall. Values estimated from sex-, age-, and birth cohort group-stratified regression of log(adiposity variable) against log(height), adjusting for exact age. ‘Overall’ value was obtained from sex- and age-stratified regression of ln(adiposity variable) against ln(height) adjusting for exact age and birth cohort group.

The age-specific optimal height powers within each birth cohort group are presented for boys and girls in Tables 3 and 4, respectively, and graphically in Fig. 1. For both boys and girls at ages 7- and 10 years, the height powers for weight and FM standardisation, showed fairly steady incremental increases across the birth cohort groups from the 1930s until the 1970s, but then a steeper increase was observed for children born in the 1980s and 1990s (Tables 3 and 4 and Fig. 1). Amongst 13-year-old boys and girls, patterns in the height powers for weight and FM were more heterogenous by birth year compared to those observed for younger children. The height powers for weight were fairly consistent for those boys and girls born between the 1930s and those born in the 1960s, followed by a decrease in the power term from the 1970–79 birth cohort group onwards amongst girls and from the 1980–89 birth cohort group amongst the boys (Tables 3 and 4 and Fig. 1). For FM, differences in the optimal height power were observed by sex and birth cohort group (Tables 3 and 4 and Fig. 1). In general, there was evidence of a decline in the power required between the 1930s and the 1940s birth cohort groups followed by a steady increase before a very steep decline for the most recent birth cohort groups (Tables 3 and 4 and Fig. 1). Height powers needed to standardise FFM for both boys and girls generally increased with age and demonstrated an increase with increasing birth cohort group, with the exception of 13-year-old girls, among whom the power term initially increased between the 1930 and 1960 birth cohort groups, after which it remained fairly consistent (Tables 3 and 4 and Fig. 1).

**Table 3:** Age-specific height powers (and associated 95% confidence intervals) to standardise body size variables for height amongst boys, by birth cohort.

		Birth cohort group						
Age								
	Body size marker	1930–39	1940–49	1950–59	1960–69	1970–79	1980–89	1990–96
7 Years		*N* = 38,410	*N* = 52,500	*N* = 31,736	*N* = 20,890	*N* = 14,437	*N* = 12,489	*N* = 10,920
	Weight	2.20 (2.19–2.22)	2.31 (2.30–2.33)	2.42 (2.40–2.44)	2.44 (2.41–2.46)	2.52 (2.48–2.55)	2.73 (2.69–2.77)	2.82 (2.76–2.87)
	Fat mass	2.46 (2.42–2.51)	2.78 (2.73–2.82)	3.04 (2.98–3.10)	3.07 (3.00–3.15)	3.32 (3.23–3.42)	3.84 (3.73–3.95)	3.89 (3.75–4.02)
	Fat-free mass	2.12 (2.12–2.13)	2.18 (2.17–2.18)	2.24 (2.22–2.25)	2.25 (2.23–2.26)	2.27 (2.26–2.29)	2.38 (2.36–2.40)	2.45 (2.42–2.47)
10 Years		*N* = 38,838	*N* = 53,918	*N* = 31,478	*N* = 21,157	*N* = 7896	*N* = 5366	*N* = 3591
	Weight	2.40 (2.38–2.42)	2.53 (2.51–2.55)	2.64 (2.61–2.66)	2.64 (2.61–2.67)	2.75 (2.70–2.81)	3.04 (2.95–3.12)	3.13 (3.03–3.24)
	Fat mass	2.72 (2.66–2.77)	3.01 (2.96–3.07)	3.26 (3.19–3.34)	3.23 (3.14–3.32)	3.48 (3.32–3.65)	4.19 (3.98–4.41)	4.27 (4.00–4.55)
	Fat-free mass	2.29 (2.28–2.30)	2.37 (2.36–2.38)	2.43 (2.42–2.44)	2.44 (2.43–2.45)	2.49 (2.47–2.52)	2.63 (2.59–2.66)	2.71 (2.66–2.75)
13 Years		*N* = 35,292	*N* = 52,288	*N* = 32,068	*N* = 19,892	*N* = 4566	*N* = 5110	*N* = 5053
	Weight	2.63 (2.61–2.65)	2.68 (2.66–2.70)	2.75 (2.73–2.78)	2.74 (2.71–2.77)	2.87 (2.80–2.94)	2.71 (2.63–2.79)	2.70 (2.62–2.78)
	Fat mass	2.67 (2.60–2.73)	2.63 (2.57–2.69)	2.72 (2.64–2.81)	2.57 (2.47–2.67)	2.90 (2.66–3.13)	2.29 (2.05–2.53)	2.08 (1.83–2.33)
	Fat-free mass	2.61 (2.60–2.62)	2.68 (2.67–2.69)	2.73 (2.72–2.74)	2.75 (2.74–2.77)	2.83 (2.79–2.86)	2.80 (2.76–2.83)	2.84 (2.81–2.88)

**Table 4:** Age-specific height powers (and associated 95% confidence intervals) to standardise body size variables for height amongst girls, by birth cohort.

		Birth cohort group						
Age								
	Body size marker	1930–39	1940–49	1950–59	1960–69	1970–79	1980–89	1990–96
7 Years		*N* = 34,385	*N* = 51,234	*N* = 31,162	*N* = 20,928	*N* = 14,070	*N* = 11,728	*N* = 10,421
	Weight	2.28 (2.26–2.30)	2.40 (2.39–2.42)	2.49 (2.47–2.51)	2.54 (2.51–2.57)	2.59 (2.55–2.63)	2.82 (2.77–2.86)	2.92 (2.87–2.97)
	Fat mass	2.58 (2.53–2.63)	2.90 (2.86–2.95)	3.09 (3.04–3.15)	3.20 (3.13–3.28)	3.35 (3.25–3.44)	3.85 (3.73–3.96)	3.93 (3.80–4.06)
	Fat-free mass	2.16 (2.15–2.18)	2.22 (2.22–2.23)	2.27 (2.26–2.29)	2.30 (2.28–2.32)	2.31 (2.29–2.33)	2.42 (2.40–2.45)	2.51 (2.48–2.53)
10 Years		*N* = 37,937	*N* = 54,080	*N* = 31,304	*N* = 21,512	*N* = 7936	*N* = 5240	*N* = 3505
	Weight	2.48 (2.46–2.50)	2.61 (2.58–2.63)	2.68 (2.66–2.71)	2.73 (2.70–2.76)	2.83 (2.77–2.89)	3.18 (3.09–3.26)	3.19 (3.08–3.30)
	Fat mass	2.90 (2.84–2.95)	3.15 (3.09–3.20)	3.30 (3.23–3.37)	3.39 (3.30–3.47)	3.57 (3.43–3.72)	4.36 (4.17–4.56)	4.21 (3.95–4.47)
	Fat-free mass	2.32 (2.31–2.33)	2.40 (2.39–2.41)	2.45 (2.44–2.46)	2.48 (2.47–2.50)	2.54 (2.51–2.56)	2.70 (2.66–2.73)	2.73 (2.68–2.78)
13 Years		*N* = 36,734	*N* = 52,418	*N* = 32,417	*N* = 20,115	*N* = 4529	*N* = 5237	*N* = 5169
	Weight	2.62 (2.59–2.64)	2.55 (2.53–2.58)	2.63 (2.60–2.67)	2.71 (2.67–2.75)	2.56 (2.47–2.66)	2.56 (2.46–2.66)	2.36 (2.25–2.47)
	Fat mass	2.68 (2.61–2.75)	2.37 (2.31–2.44)	2.52 (2.43–2.61)	2.66 (2.54–2.78)	2.24 (1.98–2.50)	2.12 (1.85–2.38)	1.37 (1.09–1.65)
	Fat-free mass	2.59 (2.58–2.60)	2.61 (2.60–2.62)	2.66 (2.65–2.68)	2.72 (2.70–2.73)	2.66 (2.62–2.70)	2.69 (2.65–2.74)	2.69 (2.64–2.74)

Sensitivity analyses testing the statistical significance of birth cohort, age and sex differences in the optimal height powers for weight, FM, and FFM were found to be highly significant at the 1% level (data not shown). Moreover, analyses exploring patterns in the optimal height powers by decade of measurement year were consistent with those observed from the main presentation by birth cohort group (Supplementary Fig. 3).

### Correlation between height and a range of weight, FM, and FFM indices

Sex- and age-specific correlation coefficients between height and a range of indices of weight, FM and FFM are presented, by birth cohort and overall, in Supplementary Tables 5 and 6. The correlation coefficients between each index and height varied by birth cohort and age. For weight, when a height power term of 2 was chosen (i.e. BMI), there was a moderate positive correlation across all birth cohort groups which increased with time. However, a weight-for-height index using a power of 3 (i.e. ponderal index) had a strong negative correlation with height in the earlier birth cohort groups which then reduced in the more recent birth cohort groups. For FM, a power of 3 resulted in the lowest correlation with height in the earlier birth cohort groups, but not in the latter groups where a power closer to 4 was required to remove the correlation with height (Supplementary Tables 5 and 6).

### Ethnic-specific optimal height powers

**Fig. 2:**
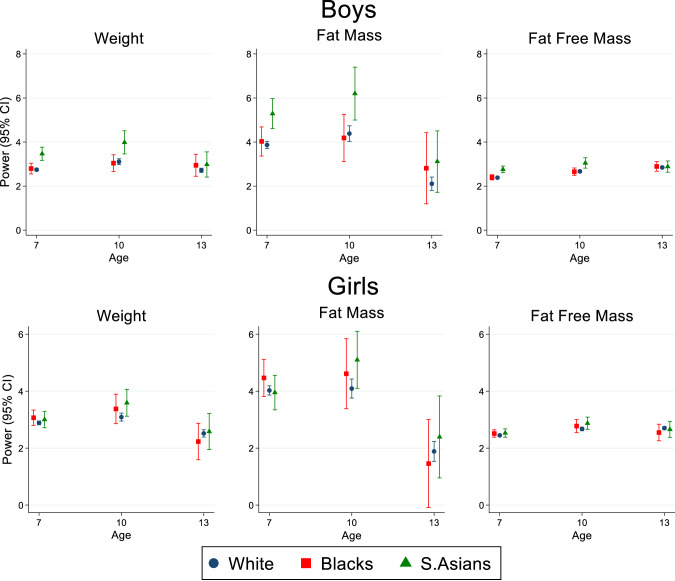
Sex- and ethnic-specific height powers (and associated 95% confidence intervals) to standardise body size variables for height in children born between 1990 and 1996 of White European, Black or South Asian ethnic origins, by age.

In the 1990–96 birth cohort group, we quantified optimal height powers needed to standardise each marker for White, Black and South Asian children separately (Fig. 2 and Supplementary Tables 11 and 12). Amongst South Asian children, there was a suggestion that the optimal height power needed to standardise the three body size markers at all three ages was higher than the power needed for White children, with the exception of FM among 7-year-old girls and FFM at 13-year-old girls, in whom the height powers were very similar in both ethnic groups (Fig. 2 and Supplementary Tables 11 and 12). Amongst Black children aged 7 and 10 years, the optimal powers were similar to those observed for White children. However, at 13 years height powers for all three markers were systematically higher amongst Black boys and lower amongst Black girls, compared to their White peers (Fig. 2 and Supplementary Tables 11 and 12).

## Discussion

### Summary of principal findings

In this study of Copenhagen schoolchildren aged 7 years, 10 years and 13 years born between 1930 and 1996, we derive for the first time the optimal height powers needed to standardise childhood FM and FFM, and demonstrate how they vary across birth cohort groups, by sex and age (within birth cohort groups), and by ethnic groups (for those born in the 1990s). The findings are also complemented by results demonstrating the variation in the optimal height powers needed to standardise childhood weight by birth year, sex and age.

For FM, FFM, and weight, the optimal height powers at 7 years and 10 years increased with birth year and were typically higher for girls compared to boys. At 13 years the height powers for all three markers initially increased with birth year before declining for those born in the more recent birth cohort groups. This decline was more marked for FM and weight compared to FFM. Furthermore, for children born in the 1990s, we observed that a greater power was needed to standardise all three markers for height for South Asian children compared to White European and Black children, albeit with low levels of precision accompanying the estimates.

These changes in the sex- and age-specific optimal powers by birth year, particularly for weight and FM, are largely explained by the much greater variation in these markers in recent birth cohort groups compared to the earlier birth cohorts. Moreover, differences observed in the optimal powers for all three markers by sex, age, and ethnicity, within birth cohort groups, can be explained by both differences across these subgroups in the variability (i.e. spread) of the markers, and their correlations with height, both of which contribute to the estimation of the optimal height powers required for standardisation.

### Comparison with other studies

Several studies have investigated the suitability of BMI as a height-independent marker of childhood adiposity and the optimal height power required to standardise weight for height in childhood [4, 6, 9, 19–21]. Most investigated how the power varied by childhood age. The study by Johnson et al. [6], while primarily investigating the differences in the power by age across the life course, also explored differences by birth year, primarily for 11-year-olds, within several historic and contemporary UK birth cohorts. Our results are generally consistent with those of Johnson et al, which found that the height power needed to standardise weight increased with birth year amongst 11-year-olds. The height powers amongst boys were estimated at 2.44 for those born in 1946, 2.61 for those born in 1958, and 3.11 for those born in 2001 and amongst girls at 2.58, 2.69, and 3.12 for those born in 1946, 1958, and 2001, respectively [6]. The same study found that amongst 10-year-old UK children born in 1970, the height powers were estimated to be 2.20 and 2.36 for boys and girls respectively [6], estimates which were lower than those observed in our study among Copenhagen children (boys: 2.75, girls: 2.83), which may be explained by the UK study containing children born only in 1970 as opposed to our results being for those born in 1970–79. Another study conducted in the 1970s with weight and height measurements of 7-year-old US children (i.e. those born in the late 1960s) [19] also found estimates of the height power similar to those of our study. Our findings pertaining to the age- and sex-specific differences in the height power for weight were also largely consistent with a study of children from Hong Kong who were born in the 1970s [4], and a study of Japanese children born in the 1980s and 1990s [21].

Far fewer studies have investigated the optimal height powers for FM and FFM, and their variability by age and sex, with no studies investigating changes in the powers over time. Our earlier studies based on UK children born in the 1990s and early 2000s estimated the height power to standardise FM to be approximately four for 7-year-olds and at least five for 10-year-olds [22, 23], which although slightly higher, are generally consistent with those of the current study. Furthermore, a study of 7-year-old children in the UK-based Millennium Cohort Study born in 2000 estimated the height powers for FM to be 3.88 and 4.31 amongst boys and girls respectively, and for FFM to be 2.43 for both boys and girls [24]. These results are consistent with our findings for children of the same age born between 1990 and 1996.

### Strengths and limitations

The key strengths of this study are the extremely large sample size of children with measured weights and heights, which allowed optimal height powers to be estimated with high levels of precision within sub-groups, and the time span of the cohort which contains childhood surveillance data across several decades spanning across the time periods both before and during the rise of the childhood obesity epidemic. Data from the CSHRR are currently available on children measured until 2011 and therefore we were not able to include very recent measurements on children from Copenhagen. However, the variability in the optimal height power observed at each of the ages for birth years 1930–1996, covering both the periods before and during the childhood obesity epidemic, highlights the difficulties of using a uniform power of height to standardise both weight and FM. Additionally, given that the CSHRR contains almost every schoolchild in Copenhagen, Denmark born between 1930 and 1996, representativeness is strong [15]. Weight and height were measured by trained physicians and nurses in a consistent manner, thus reducing the risk of measurement error. Whilst the results of this study pertaining to FM and FFM rely on the validity and accuracy in FFM estimation from the prediction equation, its high accuracy has previously been documented within several childhood settings across a range of birth years from the late 1960s onwards [16–18, 25, 26]. The prediction equation models the relationship between the included predictors and log-transformed FFM, which are unlikely to change by birth year, thus indicating the suitability of the equation also for the earlier birth years used in this study. FM estimates obtained from the prediction model have also been shown to be as accurate as those from dual-energy X-ray absorptiometry and bioelectrical impedance [18]. Limitations of this study include missing ethnicity information on children measured prior to the late 1990s onwards and thus for the estimation of childhood FFM and FM, ethnic origins were presumed to be White European. This is unlikely to have impacted the estimation of the height power in the earlier birth cohort groups, due to the low levels of immigration to Denmark in this time period [15], though estimates from the 1970–79 and 1980–89 birth cohorts may have been affected by potential misclassification of ethnic origins. Moreover, information on genetic and socioeconomic factors to explain ethnic differences was not available so we could not assess its effects on the findings.

### Implications

Height-standardised indices of weight and FM, as they remain correlated with height to varying degrees by sex, age, birth year and ethnic groups, have a different meaning within each of these groups making them difficult to interpret accurately. Given that the rationale for the standardisation of body size markers for height is to allow for meaningful comparisons within- and between individuals, this represents an important challenge. For example, the correlation between BMI and height amongst 7-year-old boys was 0.14 for those born in the 1930s but was 0.30 for boys born in the 1990s. That implies that the amount of variation in BMI explained by height was 2% in 7-year-old boys born in the 1930s but was 9% for boys born in the 1990s, highlighting the changing meaning of BMI for children born in the two different time periods, before and after the emergence of the obesity epidemic, and thus the difficulty in drawing comparisons across individuals of the same sex and age based on widely used height-standardised body size indices. Moreover, current definitions of weight status groups (underweight, healthy weight, overweight or obesity) are based upon reference values of childhood BMI, which (as demonstrated in these findings) uses an inaccurate fixed height power of 2 to standardise weight for all children irrespective of their sex, age and birth year. This is problematic as it is likely that the use of any index adopting a fixed height power to define these groups (e.g. BMI) will result in incomparable groups due to the variability in the relationship between BMI and height across subgroups. Adopting different height powers for each subgroup of sex, age and birth year would not be feasible for clinical or public health practice as this would essentially mean different adiposity markers are adopted for each subgroup of the childhood population. While some studies have suggested that body size indices should not be completely height-independent [9, 27], the issue of variability over birth years (and by sex, age, and ethnicity) in the correlation between such indices and height would remain a concern even if this were the case.

While there is a need for a shift in childhood adiposity assessment away from weight-based markers and towards fact-based assessment, particularly in light of the World Health Organisation’s recent definition of obesity as “excessive fat accumulation that presents a risk to health” [2], our results suggest that indices of FM may well be prone to the same limitations of poor height-standardisation as weight-for-height indices such as BMI. Therefore, alternative methods for taking account of height for epidemiological analyses are needed. One alternative to forming such body size indices would be to retain the original childhood body size marker (e.g. FM or weight) and take account of height in the modelling process for the analyses of interest, for example, by assessing height-adjusted mean levels of childhood FM or weight, quantifying trends over time in childhood FM or weight stratified by levels of height (e.g. quintiles), or quantifying cross-sectional or longitudinal associations between childhood FM or weight and short- or long-term outcomes of interest, adjusting for/ stratified by height.

### Future research

Future work will seek to investigate alternative approaches to interpreting FM- or weight-based assessments on an individual level, such as in clinal practice, namely whether childhood FM (along with weight, height, sex and other demographics) can be used to obtain an accurate probability of an individual developing adiposity-related health conditions in the short- and long-term. If so, such risk probabilities could be used for improved interpretation of an individual’s FM level which is indicative of associated health risks.

## Supplementary information

Supplementary Material

## Data Availability

The data material contains sensitive information and can therefore not be made publicly available. Inquiries about secure access to the CSHRR can be directed to the CSHRR steering committee (CSHRR@regionh.dk) at The Center for Clinical Research and Prevention, Copenhagen University Hospital—Bispebjerg and Frederiksberg, Capital Region, Denmark that governs the use of these data.
