# Supplementary material for: Varying optimal power for height-standardisation of childhood weight, fat mass and fat-free mass across the obesity epidemic
Source: Int J Obes (Lond). 2024 Sep 3;49(1):84–92. doi: 10.1038/s41366-024-01619-y (PMC11682999; doi:10.1038/s41366-024-01619-y)
Supplement: Supplementary file 1 — Supplementary Material [file 41366_2024_1619_MOESM1_ESM.docx]

**
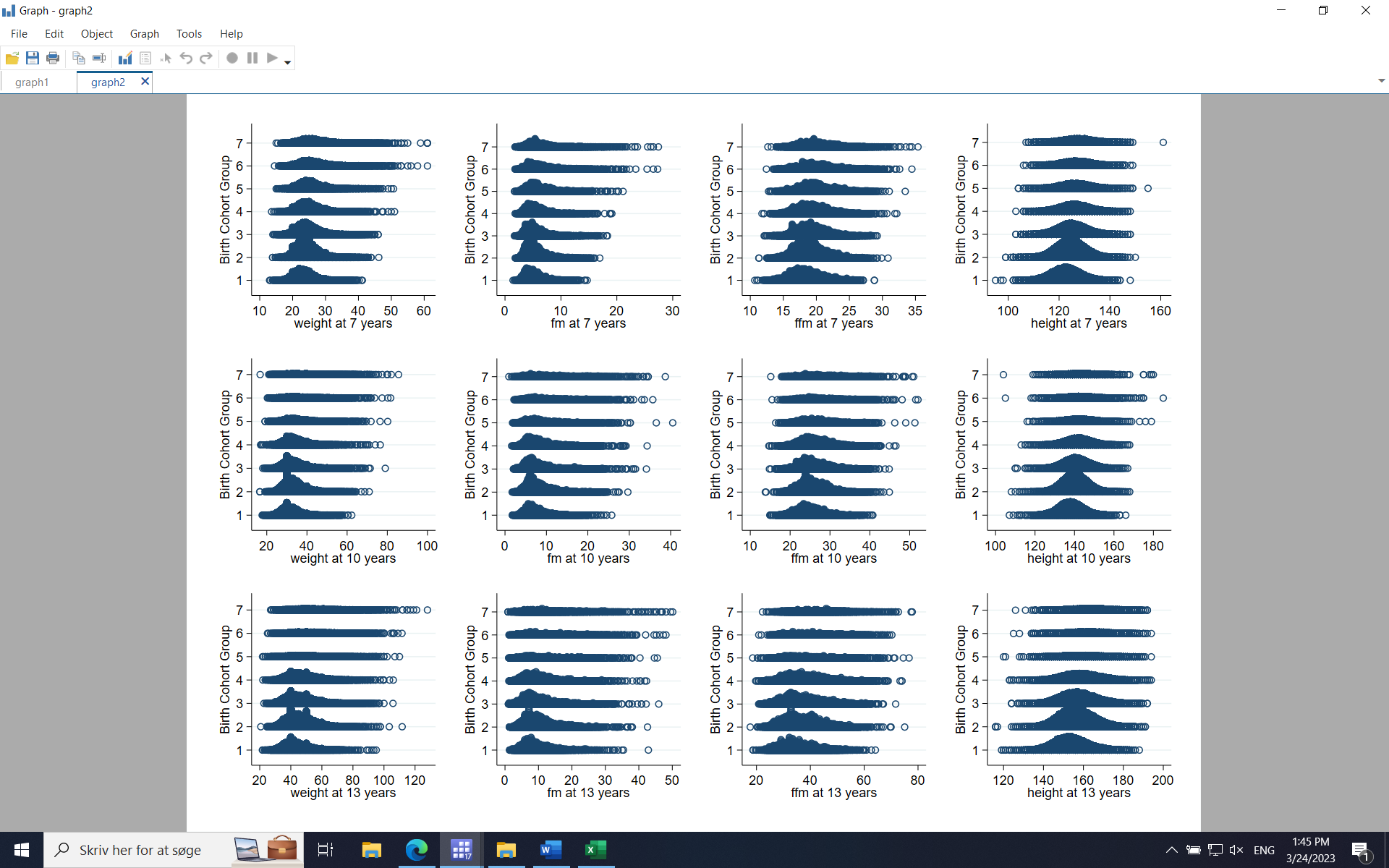
Supplementary Figure1: Histograms of weight, fat mass, fat free mass and height amongst boys at 7, 10 and 13 years, by birth cohort group**

Footnote: FM = fat mass, FFM = fat free mass. Group 1 – 1930-39, Group 2 – 1940-49, Group 3 – 1950-59, Group 4 – 1960-69, Group 5 – 1970-79, Group 6 – 1980-89, Group 7 – 1990-96


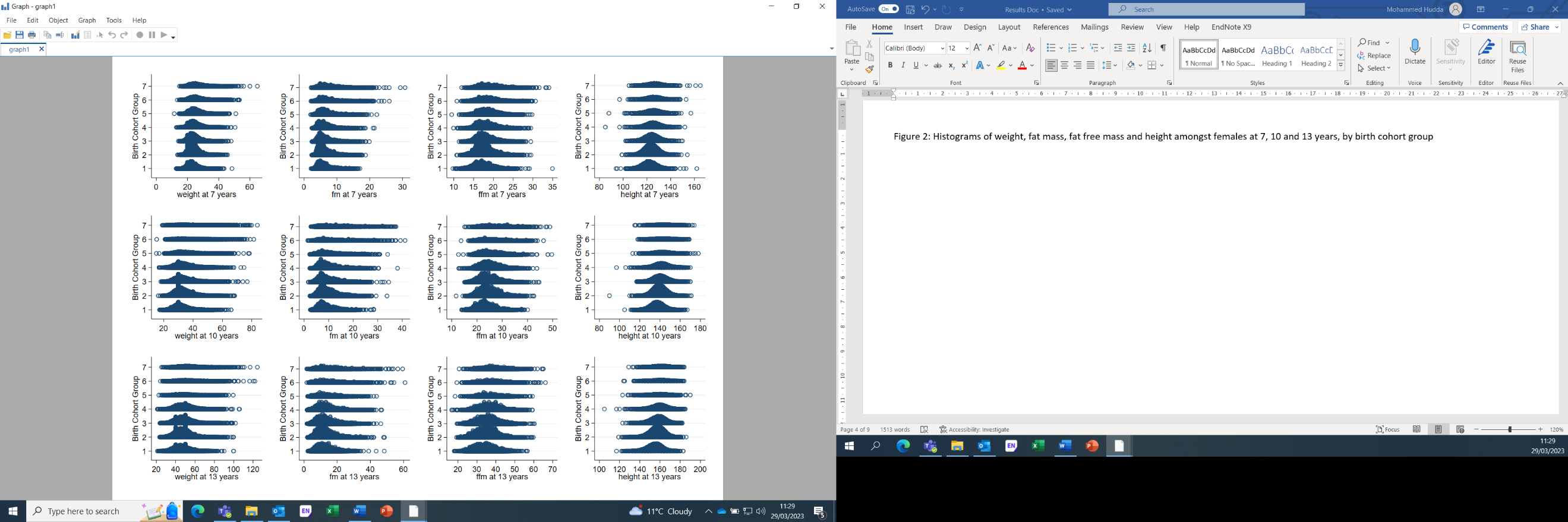
**Supplementary Figure2: Histograms of weight, fat mass, fat free mass and height amongst girls at 7, 10 and 13 years, by birth cohort group**

Footnote: FM = fat mass, FFM = fat free mass. Group 1 – 1930-39, Group 2 – 1940-49, Group 3 – 1950-59, Group 4 – 1960-69, Group 5 – 1970-79, Group 6 – 1980-89, Group 7 – 1990-96

**Supplementary Figure3: Sex- and age-specific height powers to standardise body size variables for height, by decade of measurement year and overall**

**GIRLS**

**BOYS**


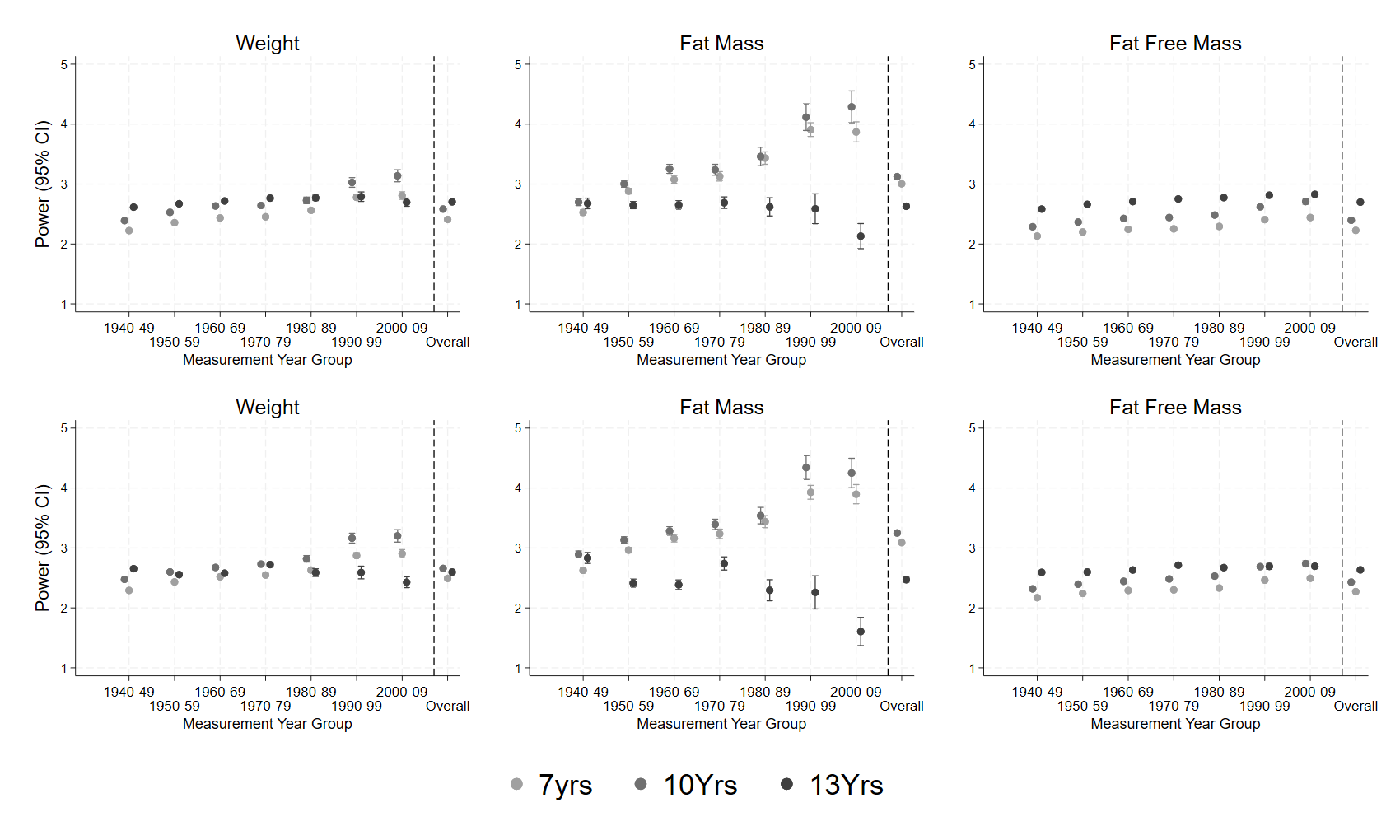

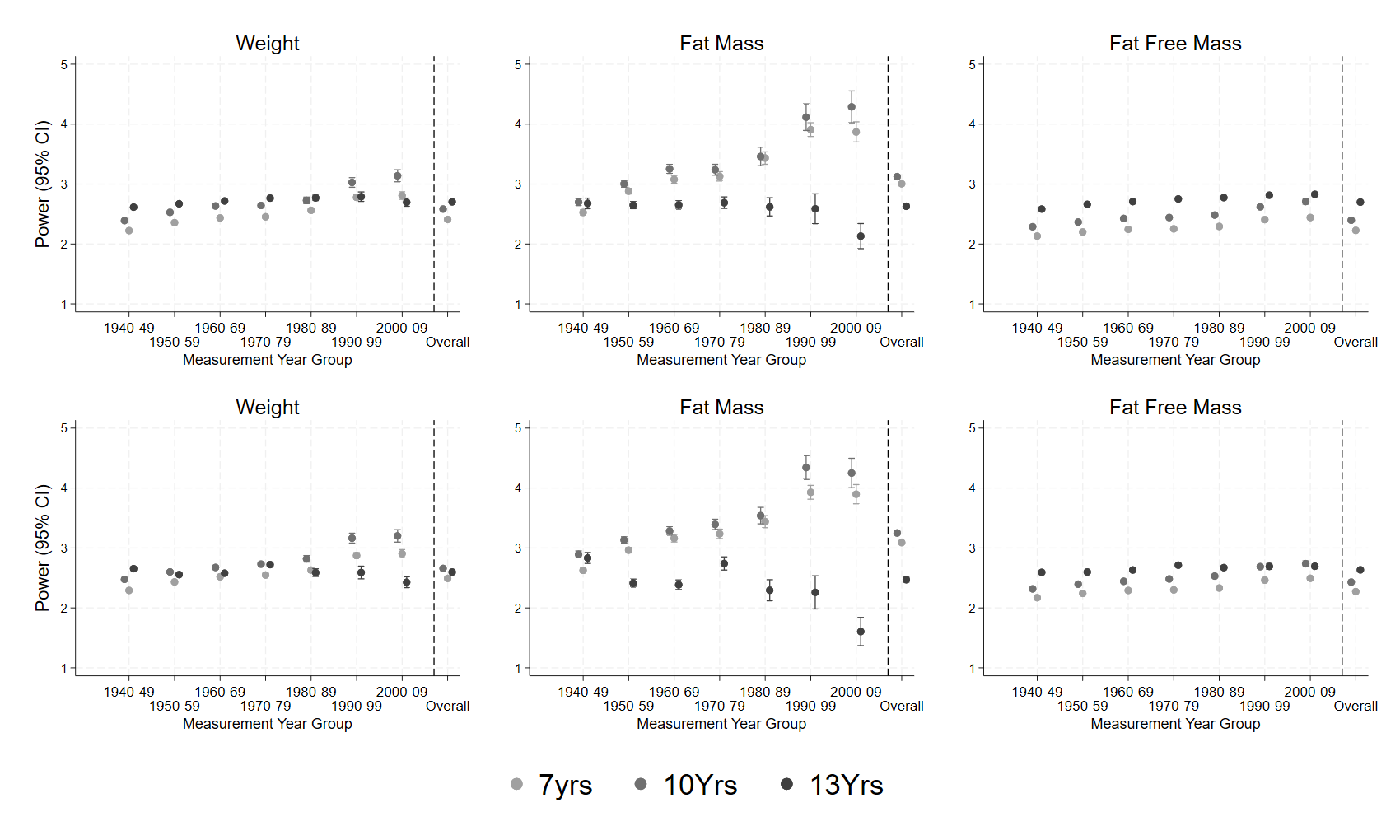


**Supplementary Table1: Standard deviation of log-transformed body size markers amongst boys at 7, 10 and 13 years, by birth cohort group**

|  | **Birth Cohort Group** | | | | | | |
| --- | --- | --- | --- | --- | --- | --- | --- |
| Age (years)  Body size marker | 1930-39 | 1940-49 | 1950-59 | 1960-69 | 1970-79 | 1980-89 | 1990-96 |
| 7 Years | *N= 38410* | *N= 52500* | *N= 31736* | *N= 20890* | *N= 14437* | *N= 12489* | *N= 10920* |
| Log(Fat Mass) | 0.24 | 0.25 | 0.27 | 0.27 | 0.29 | 0.32 | 0.34 |
| Log(Fat Free Mass) | 0.10 | 0.10 | 0.11 | 0.11 | 0.11 | 0.12 | 0.12 |
| Log(Weight) | 0.12 | 0.13 | 0.13 | 0.14 | 0.14 | 0.16 | 0.17 |
| Log(Height) | 0.05 | 0.04 | 0.04 | 0.04 | 0.04 | 0.04 | 0.04 |
|  |  |  |  |  |  |  |  |
| 10 Years | *N= 38838* | *N= 53918* | *N= 31478* | *N= 21157* | *N= 7896* | *N= 5366* | *N= 3591* |
| Log(Fat Mass) | 0.28 | 0.31 | 0.33 | 0.33 | 0.38 | 0.44 | 0.45 |
| Log(Fat Free Mass) | 0.11 | 0.11 | 0.12 | 0.12 | 0.13 | 0.14 | 0.15 |
| Log(Weight) | 0.14 | 0.15 | 0.16 | 0.16 | 0.18 | 0.21 | 0.22 |
| Log(Height) | 0.04 | 0.04 | 0.04 | 0.05 | 0.05 | 0.05 | 0.05 |
|  |  |  |  |  |  |  |  |
| 13 Years | *N= 35292* | *N= 52288* | *N= 32068* | *N= 19892* | *N= 4566* | *N= 5110* | *N= 5053* |
| Log(Fat Mass) | 0.36 | 0.38 | 0.42 | 0.41 | 0.50 | 0.48 | 0.50 |
| Log(Fat Free Mass) | 0.15 | 0.15 | 0.16 | 0.16 | 0.18 | 0.17 | 0.17 |
| Log(Weight) | 0.17 | 0.18 | 0.19 | 0.19 | 0.22 | 0.21 | 0.22 |
| Log(Height) | 0.05 | 0.05 | 0.05 | 0.06 | 0.06 | 0.06 | 0.06 |

**Supplementary Table2: Standard deviation of log-transformed body size markers amongst girls at 7, 10 and 13 years, by birth cohort group**

|  | **Birth Cohort Group** | | | | | | |
| --- | --- | --- | --- | --- | --- | --- | --- |
| Age (years)  Body size marker | 1930-39 | 1940-49 | 1950-59 | 1960-69 | 1970-79 | 1980-89 | 1990-96 |
| 7 Years | *N= 34385* | *N= 51234* | *N= 31162* | *N= 20928* | *N= 14070* | *N= 11728* | *N= 10421* |
| Log(Fat Mass) | 0.24 | 0.25 | 0.26 | 0.27 | 0.28 | 0.32 | 0.34 |
| Log(Fat Free Mass) | 0.11 | 0.11 | 0.11 | 0.11 | 0.11 | 0.12 | 0.13 |
| Log(Weight) | 0.13 | 0.14 | 0.14 | 0.15 | 0.15 | 0.17 | 0.18 |
| Log(Height) | 0.05 | 0.04 | 0.04 | 0.04 | 0.04 | 0.04 | 0.04 |
|  |  |  |  |  |  |  |  |
| 10 Years | *N= 37937* | *N= 54080* | *N= 31304* | *N= 21512* | *N= 7936* | *N= 5240* | *N= 3505* |
| Log(Fat Mass) | 0.28 | 0.31 | 0.33 | 0.34 | 0.36 | 0.43 | 0.45 |
| Log(Fat Free Mass) | 0.12 | 0.12 | 0.13 | 0.13 | 0.14 | 0.16 | 0.16 |
| Log(Weight) | 0.15 | 0.16 | 0.17 | 0.17 | 0.19 | 0.22 | 0.24 |
| Log(Height) | 0.05 | 0.05 | 0.05 | 0.05 | 0.05 | 0.05 | 0.05 |
|  |  |  |  |  |  |  |  |
| 13 Years | *N= 36734* | *N= 52418* | *N= 32417* | *N= 20115* | *N= 4529* | *N= 5237* | *N= 5169* |
| Log(Fat Mass) | 0.34 | 0.36 | 0.38 | 0.38 | 0.43 | 0.43 | 0.44 |
| Log(Fat Free Mass) | 0.13 | 0.13 | 0.13 | 0.13 | 0.14 | 0.14 | 0.14 |
| Log(Weight) | 0.17 | 0.17 | 0.17 | 0.18 | 0.20 | 0.20 | 0.19 |
| Log(Height) | 0.05 | 0.04 | 0.04 | 0.04 | 0.05 | 0.04 | 0.04 |

**Supplementary Table3: Correlation coefficients between log(height) and a range of log-transformed body size markers amongst boys aged 7, 10 and 13 years, by birth cohort group**

|  | **Birth Cohort Group** | | | | | | |
| --- | --- | --- | --- | --- | --- | --- | --- |
| Age (years)  Body size marker | 1930-39 | 1940-49 | 1950-59 | 1960-69 | 1970-79 | 1980-89 | 1990-96 |
| 7 Years | *N= 38410* | *N= 52500* | *N= 31736* | *N= 20890* | *N= 14437* | *N= 12489* | *N= 10920* |
| Fat Mass (kg) | 0.49 | 0.50 | 0.51 | 0.50 | 0.51 | 0.52 | 0.49 |
| Fat Free Mass (kg) | 0.93 | 0.92 | 0.92 | 0.92 | 0.91 | 0.90 | 0.88 |
| Weight (kg) | 0.81 | 0.80 | 0.79 | 0.79 | 0.77 | 0.76 | 0.74 |
|  |  |  |  |  |  |  |  |
| 10 Years | *N= 38838* | *N= 53918* | *N= 31478* | *N= 21157* | *N= 7896* | *N= 5366* | *N= 3591* |
| Fat Mass (kg) | 0.43 | 0.42 | 0.43 | 0.43 | 0.42 | 0.46 | 0.46 |
| Fat Free Mass (kg) | 0.93 | 0.92 | 0.92 | 0.92 | 0.91 | 0.90 | 0.89 |
| Weight (kg) | 0.79 | 0.75 | 0.75 | 0.76 | 0.73 | 0.72 | 0.71 |
|  |  |  |  |  |  |  |  |
| 13 Years | *N= 35292* | *N= 52288* | *N= 32068* | *N= 19892* | *N= 4566* | *N= 5110* | *N= 5053* |
| Fat Mass (kg) | 0.38 | 0.35 | 0.34 | 0.34 | 0.33 | 0.25 | 0.22 |
| Fat Free Mass (kg) | 0.95 | 0.95 | 0.95 | 0.95 | 0.94 | 0.92 | 0.91 |
| Weight (kg) | 0.82 | 0.80 | 0.79 | 0.81 | 0.77 | 0.70 | 0.69 |

**Supplementary Table4: Correlation coefficients between log(height) and a range of log-transformed body size markers amongst girls aged 7, 10 and 13 years, by birth cohort group**

|  | **Birth Cohort Group** | | | | | | |
| --- | --- | --- | --- | --- | --- | --- | --- |
| Age (years)  Body size marker | 1930-39 | 1940-49 | 1950-59 | 1960-69 | 1970-79 | 1980-89 | 1990-96 |
| 7 Years | *N= 34385* | *N= 51234* | *N= 31162* | *N= 20928* | *N= 14070* | *N= 11728* | *N= 10421* |
| Fat Mass (kg) | 0.51 | 0.51 | 0.53 | 0.52 | 0.53 | 0.54 | 0.52 |
| Fat Free Mass (kg) | 0.91 | 0.90 | 0.90 | 0.90 | 0.90 | 0.88 | 0.87 |
| Weight (kg) | 0.78 | 0.77 | 0.77 | 0.76 | 0.76 | 0.74 | 0.73 |
|  |  |  |  |  |  |  |  |
| 10 Years | *N= 37937* | *N= 54080* | *N= 31304* | *N= 21512* | *N= 7936* | *N= 5240* | *N= 3505* |
| Fat Mass (kg) | 0.47 | 0.46 | 0.46 | 0.46 | 0.48 | 0.52 | 0.49 |
| Fat Free Mass (kg) | 0.92 | 0.91 | 0.91 | 0.91 | 0.91 | 0.90 | 0.89 |
| Weight (kg) | 0.76 | 0.74 | 0.74 | 0.74 | 0.74 | 0.73 | 0.71 |
|  |  |  |  |  |  |  |  |
| 13 Years | *N= 36734* | *N= 52418* | *N= 32417* | *N= 20115* | *N= 4529* | *N= 5237* | *N= 5169* |
| Fat Mass (kg) | 0.37 | 0.30 | 0.29 | 0.30 | 0.25 | 0.21 | 0.14 |
| Fat Free Mass (kg) | 0.92 | 0.91 | 0.90 | 0.90 | 0.88 | 0.85 | 0.84 |
| Weight (kg) | 0.73 | 0.68 | 0.67 | 0.68 | 0.62 | 0.57 | 0.52 |

**Supplementary Table5: Correlation coefficients between height and a range of body size indices amongst boys aged 7, 10 and 13 years, by birth cohort group and overall**

| Age (years) Body size indices | **Birth Cohort Group** | | | | | | | |
| --- | --- | --- | --- | --- | --- | --- | --- | --- |
|  | 1930-39 | 1940-49 | 1950-59 | 1960-69 | 1970-79 | 1980-89 | 1990-96 | *Overall* |
| 7 Years |  |  |  |  |  |  |  |  |
| Body Mass Index (kg/m^2^) | 0.14 | 0.19 | 0.23 | 0.23 | 0.24 | 0.30 | 0.30 | 0.24 |
| Ponderal Index (kg/m^3^) | -0.43 | -0.34 | -0.28 | -0.28 | -0.22 | -0.10 | -0.06 | -0.26 |
| Fat Mass Index (kg/m^2^) | 0.13 | 0.18 | 0.22 | 0.22 | 0.24 | 0.29 | 0.27 | 0.24 |
| Fat Mass Index (kg/m^3^) | -0.08 | -0.01 | 0.05 | 0.04 | 0.08 | 0.16 | 0.15 | 0.07 |
| Fat Mass Index (kg/m^4^) | -0.28 | -0.20 | -0.14 | -0.14 | -0.09 | 0.01 | 0.02 | -0.12 |
| Fat Free Mass Index (kg/m^2^) | 0.15 | 0.19 | 0.24 | 0.24 | 0.24 | 0.30 | 0.31 | 0.25 |
| Fat Free Mass Index (kg/m^3^) | -0.71 | -0.66 | -0.62 | -0.62 | -0.57 | -0.47 | -0.40 | -0.62 |
| 10 Years |  |  |  |  |  |  |  |  |
| Body Mass Index (kg/m^2^) | 0.22 | 0.24 | 0.27 | 0.27 | 0.28 | 0.33 | 0.34 | 0.28 |
| Ponderal Index (kg/m^3^) | -0.28 | -0.19 | -0.13 | -0.14 | -0.08 | 0.03 | 0.05 | -0.14 |
| Fat Mass Index (kg/m^2^) | 0.14 | 0.17 | 0.19 | 0.19 | 0.21 | 0.27 | 0.27 | 0.21 |
| Fat Mass Index (kg/m^3^) | -0.02 | 0.02 | 0.06 | 0.06 | 0.09 | 0.16 | 0.17 | 0.07 |
| Fat Mass Index (kg/m^4^) | -0.19 | -0.12 | -0.08 | -0.09 | -0.04 | 0.05 | 0.05 | -0.08 |
| Fat Free Mass Index (kg/m^2^) | 0.34 | 0.36 | 0.39 | 0.41 | 0.40 | 0.44 | 0.46 | 0.40 |
| Fat Free Mass Index (kg/m^3^) | -0.60 | -0.51 | -0.46 | -0.46 | -0.38 | -0.25 | -0.19 | -0.48 |
| 13 Years |  |  |  |  |  |  |  |  |
| Body Mass Index (kg/m^2^) | 0.33 | 0.32 | 0.32 | 0.34 | 0.32 | 0.23 | 0.22 | 0.34 |
| Ponderal Index (kg/m^3^) | -0.18 | -0.15 | -0.11 | -0.11 | -0.05 | -0.10 | -0.10 | -0.11 |
| Fat Mass Index (kg/m^2^) | 0.11 | 0.09 | 0.10 | 0.09 | 0.11 | 0.03 | 0.00 | 0.11 |
| Fat Mass Index (kg/m^3^) | -0.05 | -0.05 | -0.03 | -0.05 | -0.01 | -0.08 | -0.10 | -0.03 |
| Fat Mass Index (kg/m^4^) | -0.20 | -0.19 | -0.16 | -0.18 | -0.12 | -0.18 | -0.19 | -0.16 |
| Fat Free Mass Index (kg/m^2^) | 0.62 | 0.62 | 0.63 | 0.66 | 0.62 | 0.55 | 0.55 | 0.64 |
| Fat Free Mass Index (kg/m^3^) | -0.39 | -0.31 | -0.24 | -0.23 | -0.13 | -0.14 | -0.10 | -0.27 |

**Supplementary Table6: Correlation coefficients between height and a range of adiposity indices amongst girls aged 7, 10 and 13 years, by birth cohort group and overall**

| Age (years) Body size indices | Birth Cohort Group | | | | | | | |
| --- | --- | --- | --- | --- | --- | --- | --- | --- |
|  | 1930-39 | 1940-49 | 1950-59 | 1960-69 | 1970-79 | 1980-89 | 1990-96 | *Overall* |
| 7 Years |  |  |  |  |  |  |  |  |
| Body Mass Index (kg/m^2^) | 0.17 | 0.21 | 0.24 | 0.24 | 0.25 | 0.30 | 0.31 | 0.26 |
| Ponderal Index (kg/m^3^) | -0.35 | -0.26 | -0.23 | -0.20 | -0.17 | -0.06 | -0.02 | -0.20 |
| Fat Mass Index (kg/m^2^) | 0.16 | 0.21 | 0.23 | 0.23 | 0.25 | 0.30 | 0.30 | 0.25 |
| Fat Mass Index (kg/m^3^) | -0.05 | 0.02 | 0.05 | 0.06 | 0.08 | 0.16 | 0.17 | 0.08 |
| Fat Mass Index (kg/m^4^) | -0.05 | 0.02 | 0.05 | 0.06 | 0.08 | 0.16 | 0.17 | 0.08 |
| Fat Free Mass Index (kg/m^2^) | 0.17 | 0.21 | 0.24 | 0.25 | 0.26 | 0.30 | 0.33 | 0.26 |
| Fat Free Mass Index (kg/m^3^) | -0.64 | -0.58 | -0.56 | -0.53 | -0.52 | -0.41 | -0.34 | -0.55 |
| 10 Years |  |  |  |  |  |  |  |  |
| Body Mass Index (kg/m^2^) | 0.23 | 0.25 | 0.27 | 0.28 | 0.30 | 0.36 | 0.34 | 0.28 |
| Ponderal Index (kg/m^3^) | -0.22 | -0.15 | -0.12 | -0.10 | -0.06 | 0.06 | 0.06 | -0.11 |
| Fat Mass Index (kg/m^2^) | 0.17 | 0.19 | 0.20 | 0.21 | 0.24 | 0.30 | 0.28 | 0.22 |
| Fat Mass Index (kg/m^3^) | 0.00 | 0.03 | 0.06 | 0.07 | 0.10 | 0.18 | 0.16 | 0.07 |
| Fat Mass Index (kg/m^4^) | 0.00 | 0.03 | 0.06 | 0.07 | 0.10 | 0.18 | 0.16 | 0.07 |
| Fat Free Mass Index (kg/m^2^) | 0.33 | 0.36 | 0.39 | 0.41 | 0.43 | 0.47 | 0.47 | 0.40 |
| Fat Free Mass Index (kg/m^3^) | -0.54 | -0.46 | -0.43 | -0.40 | -0.36 | -0.21 | -0.18 | -0.44 |
| 13 Years |  |  |  |  |  |  |  |  |
| Body Mass Index (kg/m^2^) | 0.24 | 0.19 | 0.21 | 0.22 | 0.16 | 0.14 | 0.09 | 0.21 |
| Ponderal Index (kg/m^3^) | -0.13 | -0.14 | -0.11 | -0.08 | -0.12 | -0.11 | -0.14 | -0.12 |
| Fat Mass Index (kg/m^2^) | 0.10 | 0.04 | 0.06 | 0.07 | 0.03 | 0.01 | -0.05 | 0.07 |
| Fat Mass Index (kg/m^3^) | -0.04 | -0.08 | -0.05 | -0.04 | -0.08 | -0.08 | -0.14 | -0.05 |
| Fat Mass Index (kg/m^4^) | -0.04 | -0.08 | -0.05 | -0.04 | -0.08 | -0.08 | -0.14 | -0.05 |
| Fat Free Mass Index (kg/m^2^) | 0.49 | 0.46 | 0.47 | 0.49 | 0.42 | 0.39 | 0.37 | 0.48 |
| Fat Free Mass Index (kg/m^3^) | -0.31 | -0.27 | -0.22 | -0.18 | -0.21 | -0.16 | -0.15 | -0.25 |

**Supplementary Table7: Summary statistics of body size variables at ages 7, 10 and 13 years amongst boys born between 1990 and 1996, by ethnic group**

|  | Median (lower quartile – upper quartile) | | | |  |
| --- | --- | --- | --- | --- | --- |
| Age (y)  Body size marker | | White | Black | South Asian | |
| 7 Years | | N = 7422 | N = 528 | N = 418 | |
| Weight (kg) | | 26.0 (23.6 - 28.8) | 26.1 (23.5 - 29.5) | 24.9 (22.0 - 28.0) | |
| Fat Mass (kg) | | 5.52 (4.58 - 6.79) | 5.35 (4.24 - 7.16) | 6.36 (5.13 - 8.20) | |
| Fat Free Mass (kg) | | 20.44 (18.98 - 21.99) | 20.69 (19.14 - 22.54) | 18.50 (16.93 - 20.16) | |
| Height (m) | | 1.28 (1.24 - 1.31) | 1.27 (1.24 - 1.31) | 1.26 (1.22 - 1.30) | |
|  | |  |  |  | |
| 10 Years | | N = 2178 | N = 235 | N = 172 | |
| Weight (kg) | | 36.0 (32.0 - 41.8) | 35.6 (31.0 - 41.6) | 35.2 (31.0 - 43.9) | |
| Fat Mass (kg) | | 8.29 (6.41 - 11.32) | 7.53 (5.58 - 11.01) | 9.62 (7.74 - 15.25) | |
| Fat Free Mass (kg) | | 27.70 (25.26 - 30.60) | 27.97 (25.30 - 31.34) | 25.41 (23.08 - 28.73) | |
| Height (m) | | 1.44 (1.39 - 1.49) | 1.44 (1.39 - 1.48) | 1.42 (1.38 - 1.47) | |
|  | |  |  |  | |
| 13 Years | | N = 3549 | N = 202 | N = 183 | |
| Weight (kg) | | 52.4 (45.6 - 60.0) | 49.6 (43.0 - 59.5) | 53.0 (44.5 - 64.2) | |
| Fat Mass (kg) | | 10.29 (7.52 - 13.94) | 9.51 (6.83 - 13.88) | 13.61 (10.49 - 22.37) | |
| Fat Free Mass (kg) | | 41.54 (37.02 - 46.55) | 39.40 (35.57 - 44.77) | 38.65 (33.87 - 43.60) | |
| Height (m) | | 1.64 (1.58 - 1.70) | 1.60 (1.55 - 1.66) | 1.62 (1.55 - 1.67) | |

**Supplementary Table8: Median and lower and upper quartiles of body size variables at ages 7, 10 and 13 years amongst girls born between 1990 and 1996, by ethnic group**

|  | Ethnic Group | | | |  |
| --- | --- | --- | --- | --- | --- |
| Age (y)  Body size marker | | White | Black | South Asian | |
| 7 Years | | N = 6985 | N = 515 | N = 442 | |
| Weight (kg) | | 25.5 (23.0 - 28.5) | 26.4 (23.2 - 30.4) | 24.1 (21.5 - 28.5) | |
| Fat Mass (kg) | | 6.30 (5.18 - 7.95) | 6.59 (5.13 - 8.91) | 6.99 (5.65 - 9.04) | |
| Fat Free Mass (kg) | | 19.15 (17.70 - 20.74) | 19.82 (18.17 - 21.74) | 17.18 (15.73 - 19.38) | |
| Height (m) | | 1.27 (1.23 - 1.30) | 1.27 (1.23 - 1.31) | 1.25 (1.21 - 1.29) | |
|  | |  |  |  | |
| 10 Years | | N = 2161 | N = 222 | N = 177 | |
| Weight (kg) | | 36.2 (31.5 - 42.8) | 37.0 (32.7 - 46.0) | 37.6 (31.1 - 45.5) | |
| Fat Mass (kg) | | 9.51 (7.31 - 13.21) | 10.10 (7.77 - 14.98) | 11.99 (8.70 - 16.85) | |
| Fat Free Mass (kg) | | 26.60 (23.96 - 29.71) | 27.31 (24.82 - 30.47) | 24.99 (22.24 - 28.17) | |
| Height (m) | | 1.44 (1.39 - 1.49) | 1.44 (1.39 - 1.49) | 1.44 (1.38 - 1.48) | |
|  | |  |  |  | |
| 13 Years | | N = 3508 | N = 241 | N = 213 | |
| Weight (kg) | | 51.0 (45.5 - 57.8) | 54.0 (47.7 - 63.3) | 49.5 (43.5 - 55.5) | |
| Fat Mass (kg) | | 12.32 (9.45 - 16.28) | 14.64 (11.13 - 20.32) | 14.71 (11.72 - 19.20) | |
| Fat Free Mass (kg) | | 38.48 (35.31 - 42.06) | 39.22 (36.13 - 43.20) | 34.10 (31.23 - 36.90) | |
| Height (m) | | 1.62 (1.58 - 1.67) | 1.60 (1.56 - 1.64) | 1.58 (1.54 - 1.62) | |

**Supplementary Table9: Correlation coefficients between log(height) and a range of body size markers (on the log-transformed scale) at ages 7, 10 and 13 years amongst boys born between 1990 and 1996, by ethnic group**

|  | Ethnic Group | | |
| --- | --- | --- | --- |
| Age (y)  Body size marker | White | Black | South Asian |
| 7 Years | *N = 7422* | *N = 528* | *N = 418* |
| Fat Mass (kg) | 0.51 | 0.47 | 0.60 |
| Fat Free Mass (kg) | 0.89 | 0.87 | 0.87 |
| Weight (kg) | 0.75 | 0.71 | 0.75 |
|  |  |  |  |
| 10 Years | *N = 2178* | *N = 235* | *N = 172* |
| Fat Mass (kg) | 0.47 | 0.45 | 0.61 |
| Fat Free Mass (kg) | 0.90 | 0.90 | 0.90 |
| Weight (kg) | 0.73 | 0.72 | 0.75 |
|  |  |  |  |
| 13 Years | *N = 3549* | *N = 202* | *N = 183* |
| Fat Mass (kg) | 0.22 | 0.24 | 0.30 |
| Fat Free Mass (kg) | 0.92 | 0.88 | 0.86 |
| Weight (kg) | 0.71 | 0.65 | 0.61 |

**Supplementary Table10: Correlation coefficients between log(height) and a range of body size markers (on the log-transformed scale) at ages 7, 10 and 13 years amongst girls born between 1990 and 1996, by ethnic group**

|  | **Ethnic Group** | | |
| --- | --- | --- | --- |
| Age (y)  Body size marker | Whites | Blacks | South Asians |
| 7 Years | *N = 6985* | *N = 515* | *N = 442* |
| Fat Mass (kg) | 0.53 | 0.51 | 0.55 |
| Fat Free Mass (kg) | 0.88 | 0.86 | 0.87 |
| Weight (kg) | 0.73 | 0.71 | 0.72 |
|  |  |  |  |
| 10 Years | *N = 2161* | *N = 222* | *N = 177* |
| Fat Mass (kg) | 0.48 | 0.45 | 0.60 |
| Fat Free Mass (kg) | 0.90 | 0.86 | 0.90 |
| Weight (kg) | 0.71 | 0.66 | 0.76 |
|  |  |  |  |
| 13 Years | *N = 3508* | *N = 241* | *N = 213* |
| Fat Mass (kg) | 0.19 | 0.13 | 0.22 |
| Fat Free Mass (kg) | 0.86 | 0.75 | 0.79 |
| Weight (kg) | 0.57 | 0.42 | 0.49 |

**Supplementary Table11: Ethnic-specific height powers (and associated 95% confidence intervals) to standardise body size variables for height in boys born between 1990 and 1996 of White European, Black or South Asian ethnic origins, by age**

|  | **Power (95% confidence interval)** | | |
| --- | --- | --- | --- |
| Age (y)  Body size marker | Whites | Blacks | South Asians |
| 7 Years | N = 7422 | N = 528 | N = 418 |
| Weight (kg) | 2.73 (2.67 - 2.79) | 2.82 (2.58 - 3.06) | 3.47 (3.17 - 3.77) |
| Fat Mass (kg) | 3.84 (3.68 - 3.99) | 4.10 (3.44 - 4.76) | 5.29 (4.61 - 5.97) |
| Fat Free Mass (kg) | 2.38 (2.35 - 2.41) | 2.41 (2.29 - 2.53) | 2.76 (2.61 - 2.91) |
|  |  |  |  |
| 10 Years | N = 2178 | N = 235 | N = 172 |
| Weight (kg) | 3.06 (2.94 - 3.19) | 3.04 (2.66 - 3.42) | 3.98 (3.46 - 4.51) |
| Fat Mass (kg) | 4.26 (3.92 - 4.60) | 4.20 (3.13 - 5.26) | 6.20 (5.00 - 7.40) |
| Fat Free Mass (kg) | 2.65 (2.59 - 2.70) | 2.66 (2.49 - 2.82) | 3.05 (2.81 - 3.28) |
|  |  |  |  |
| 13 Years | N = 3549 | N = 202 | N = 183 |
| Weight (kg) | 2.71 (2.61 - 2.80) | 2.95 (2.45 - 3.44) | 2.98 (2.41 - 3.55) |
| Fat Mass (kg) | 2.09 (1.80 - 2.39) | 2.82 (1.22 - 4.43) | 3.11 (1.72 - 4.51) |
| Fat Free Mass (kg) | 2.84 (2.80 - 2.88) | 2.89 (2.67 - 3.11) | 2.88 (2.63 - 3.14) |

**Supplementary Table12: Ethnic-specific height powers (and associated 95% confidence intervals) to standardise body size variables for height in girls born between 1990 and 1996 of White European, Black or South Asian ethnic origins, by age**

|  | **Power (95% confidence interval)** | | |
| --- | --- | --- | --- |
| Age (y)  Body size marker | Whites | Blacks | South Asians |
| 7 Years | *N = 6985* | *N = 515* | *N = 442* |
| Weight (kg) | 2.89 (2.83 - 2.96) | 3.07 (2.80 - 3.34) | 3.00 (2.71 - 3.29) |
| Fat Mass (kg) | 4.03 (3.87 - 4.18) | 4.46 (3.82 - 5.11) | 3.95 (3.35 - 4.56) |
| Fat Free Mass (kg) | 2.45 (2.42 - 2.48) | 2.51 (2.38 - 2.65) | 2.53 (2.39 - 2.68) |
|  |  |  |  |
| 10 Years | *N = 2161* | *N = 222* | *N = 177* |
| Weight (kg) | 3.10 (2.97 - 3.24) | 3.41 (2.90 - 3.92) | 3.59 (3.12 - 4.07) |
| Fat Mass (kg) | 4.11 (3.79 - 4.44) | 4.68 (3.46 - 5.90) | 5.10 (4.10 - 6.10) |
| Fat Free Mass (kg) | 2.68 (2.62 - 2.74) | 2.79 (2.56 - 3.02) | 2.88 (2.66 - 3.09) |
|  |  |  |  |
| 13 Years | *N = 3508* | *N = 241* | *N = 213* |
| Weight (kg) | 2.52 (2.40 - 2.65) | 2.26 (1.62 - 2.90) | 2.58 (1.95 - 3.22) |
| Fat Mass (kg) | 1.89 (1.56 - 2.23) | 1.52 (-0.03 - 3.06) | 2.40 (0.96 - 3.84) |
| Fat Free Mass (kg) | 2.71 (2.65 - 2.76) | 2.56 (2.27 - 2.85) | 2.66 (2.38 - 2.94) |
